# Similar Pain Intensity Reductions and Trunk Strength Improvements Following Whole-Body Electromyostimulation vs. Whole-Body Vibration vs. Conventional Back-Strengthening Training in Chronic Non-specific Low Back Pain Patients: A Three-Armed Randomized Controlled Trial

**DOI:** 10.3389/fphys.2021.664991

**Published:** 2021-04-13

**Authors:** Florian Micke, Anja Weissenfels, Nicolas Wirtz, Simon von Stengel, Ulrike Dörmann, Matthias Kohl, Heinz Kleinöder, Lars Donath, Wolfgang Kemmler

**Affiliations:** ^1^Department of Intervention Research in Exercise Training, Institute of Training Science and Sport Informatics, German Sport University Cologne, Cologne, Germany; ^2^Institute of Medical Physics, Friedrich-Alexander University of Erlangen-Nürnberg, Erlangen, Germany; ^3^Central Library for Sport Science, German Sport University Cologne, Cologne, Germany; ^4^Department of Medical and Life Sciences, University of Furtwangen, Villingen-Schwenningen, Germany

**Keywords:** lumbar spine pain, electrical stimulation, MVC, strength training, vibration training

## Abstract

The aim of this multicenter trial was to compare the effects of whole-body electromyostimulation (WB-EMS) and whole-body vibration (WBV) with conventional back-strengthening training (CT) on changes in mean back pain intensity (MPI) and trunk strength in patients suffering from chronic non-specific low back pain (CNLBP). Two-hundred and forty CNLBP patients (40–70 years; 62% female) were randomly assigned to three intervention arms (WB-EMS: *n* = 80 vs. WBV: *n* = 80 vs. CT: *n* = 80). All training intervention programs were performed for 12 weeks in their usual commercial training setting. Before and during the last 4 weeks of the intervention, MPI was recorded using a 4-week pain diary. Additionally, maximal isometric trunk extension and -flexion strength was assessed on the BackCheck® machine. A moderate but significant decrease of MPI was observed in all groups (WB-EMS: 29.7 ± 39.1% (SMD 0.50) vs. WBV: 30.3 ± 39.3% (SMD 0.57) vs. CT: 30.5 ± 39.6% (SMD 0.59); *p* < 0.001). Similar findings were observed for maximal isometric strength parameters with a significant increase in all groups (extension: WB-EMS: 17.1 ± 25.5% vs. WBV: 16.2 ± 23.6% vs. CT: 21.6 ± 27.5%; *p* < 0.001; flexion: WB-EMS: 13.3 ± 25.6% vs. WBV: 13.9 ± 24.0% vs. CT: 13.9 ± 25.4%; *p* < 0.001). No significant interaction effects for MPI (*p* = 0.920) and strength parameters (extension: *p* = 0.436; flexion: *p* = 0.937) were observed. WB-EMS, WBV, and CT are comparably effective in improving MPI and trunk strength. However, training volume of WB-EMS was 43 or 62% lower, compared with CT and WBV.

## Introduction

With a global lifetime prevalence of 38.9%, low back pain is considered one of the most impactful health issues worldwide (Hoy et al., 2012). Low back pain serves as a multifactorial disease with different underlying etiologies (e.g., lifestyle and social demographic factors, occupational factors, psychological factors, age, and gender; Manchikanti et al., 2014). Those factors create substantial disease burden on a personal, community, and financial level (Rapoport et al., 2004). Previous studies implicated that a large proportion of the reported back pain can be diagnosed as non-specific, referring to a condition that makes it difficult to identify a specific cause of the pain with an unknown pathology (Abraham and Killackey-Jones, 2002).

In the current literature, there is a large amount of studies focusing on conventional training programs and the reduction of low back pain. Researchers concentrated on strength/resistance programs (Jackson et al., 2011; Steele et al., 2013; Vincent et al., 2014), coordination/stabilization programs (Shaughnessy and Caulfield, 2004; Critchley et al., 2007; Costa et al., 2009), cardiorespiratory exercise (Kell and Asmundson, 2009; Cuesta-Vargas et al., 2011), and combined-exercise methods (Mannion et al., 2001; Nassif et al., 2011). Beside a variety of effective exercise-based training regimen, strength training has been shown to reduce pain intensity and improve physical function of patients with chronic non-specific low back pain (CNLBP). A previous meta-analysis provided evidence on pain reduction following conventional exercise (Searle et al., 2015). On the other hand, studies show that a sedentary lifestyle significantly increases the incidence of recurrent low back pain (Citko et al., 2018) and that there is an association between inactivity, low back pain, and decreased back strength (Bo Andersen et al., 2006). Patients suffering from chronic back pain often avoid exercise as a result of a feeling of susceptibility to painful injury or reinjury (Ishak et al., 2017). Furthermore, studies have been shown that besides of time restrictions (Korsch et al., 2016), CNLBP frequently report kinesiophobia (Lüning Bergsten et al., 2012) as a primary reason for being physically inactive. However, evidence is provided that exercise programs, in particular, are effective for reducing fear-avoidance behavior (Hanel et al., 2020). Consequently, time-efficient and effective training programs that can be easily performed by less active and possibly fear-avoidance individuals are needed to keep patients regularly and sustainably active.

Modern training technologies such as whole-body electromyostimulation (WB-EMS) and whole-body vibration (WBV) have been proven as effective, appealing, and time-efficient training methods in different exercise settings (Padulo et al., 2014a, 2016; Filipovic et al., 2016; Ardigò et al., 2018) and populations (Filipovic et al., 2011; Kemmler et al., 2018; Lai et al., 2018; Fischer et al., 2019). These alternative training methods gaining attention and popularity. The stimulation with WB-EMS and WBV alters the neuro-muscular pattern of muscle recruitment and leads to acute and long-term effects in performance as well as in rehabilitation (Padulo et al., 2014b; Fischer et al., 2019; Kemmler et al., 2021).

In the field of WBV, there are a few RCTs with CNLBP patients (del Pozo-Cruz et al., 2011; Wang et al., 2019) but despite one (Weissenfels et al., 2018), there are hardly any RCTs that evaluate the training effects of WB-EMS on low back pain. Due to their time-effectiveness and low (voluntary) loading characteristics, these alternative training technologies might be an alternative training option for patients suffering from CNLBP, although they are not considered as common means for treating back pain. However, studies investigating WB-EMS or WBV in the field of CNLBP are rare and randomly controlled comparative studies are missing.

Against the aforementioned background, the aim of this randomized controlled multicenter study was to compare the effects of WB-EMS and WBV with conventional back-strengthening training (CT) on mean back pain intensity and strength indices in patients with CNLBP. It was hypothesized that both WB-EMS and WBV induce similar improvements of pain intensity and trunk strength at notable shorter total training volumes compared to conventional back-strengthening training.

## Materials and Methods

### Study Design

This study was conceptualized as a three-armed randomized controlled trail with parallel-group design comparing the effects of three 12-weeks lasting training interventions applying (a) WB-EMS, (b) WBV, or (c) CT on mean low back pain intensity and trunk strength. In order to elaborate training effects, mean pain intensity (MPI), recorded using a 4-week pain diary, and maximal isometric voluntary trunk strength were measured before (baseline) and after the intervention (12-week follow-up; see Outcome Measures).

This study is part of a multicenter project, conducted by the Institute of Medical Physics of the Friedrich Alexander University Erlangen-Nürnberg (FAU) and the Department of Intervention Research in Exercise Training of the German Sport University Cologne. A preliminary two-group comparison (WB-EMS vs. CT) of this multicenter project has been described by Weissenfels et al. (2019).

The study protocol was approved by the Ethical Committee of the FAU (application no. 224_15b) and complied with the Declaration of Helsinki. The multicenter project was registered in the German Clinical Trial Register (DRKS-ID: DRKS00009528).

### Participants

**Figure 1:**
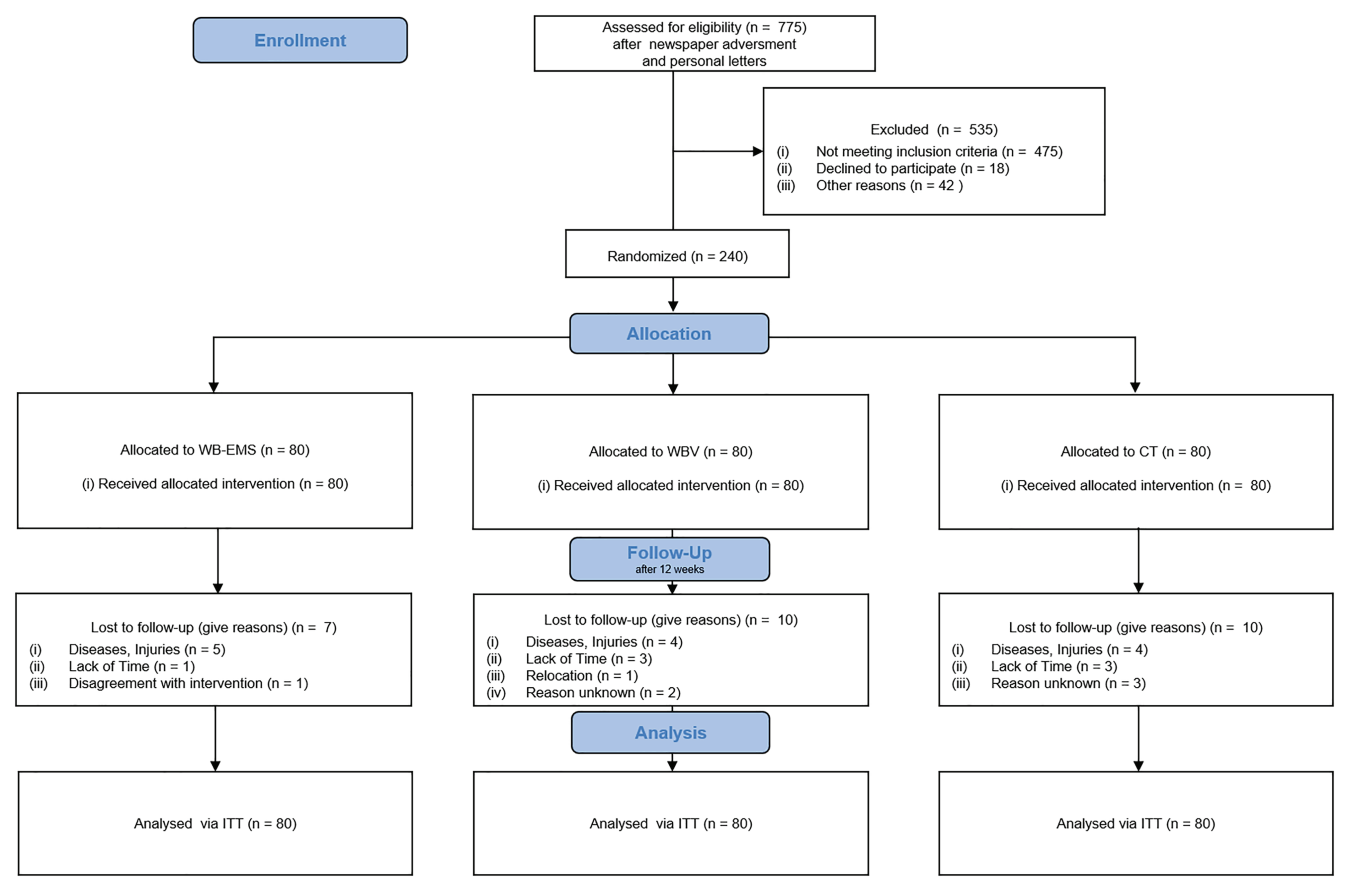
CONSORT flow diagram of the study intervention.

Two-hundred and forty middle-aged adults with non-specific low back pain were recruited to participate in the study (see Figure 1). Participants characteristics are presented in Table 1. Inclusion criteria for all participants were: (a) age between 40 and 70 years; (b) chronic pain in the lumbar spine (at least 50% of the days of the last 3 months); (c) self-report of no existing orthopedic diagnosis (non-specific type of LBP); (d) average basal pain intensity ≥1 on numeric rating scale (NRS) 0–10; (e) no frequent intake of analgesics (>4 days/week); (f) no pharmacological therapy or diseases affecting muscle metabolism (e.g., glucocorticoids); (g) no contraindications for WB-EMS or WBV application (e.g., epilepsy, cardiac pacemaker, thrombosis, and total endoprosthesis), and (h) attendance >80% of the training sessions. The participants were recruited *via* 12,000 personal letters and eight newspaper advertisements with a large local reach.

**Table 1:** Baseline characteristics of the three intervention groups.

	WB-EMS (*n* = 80)	WBV (*n* = 80)	CT (*n* = 80)
Gender (m/f)^a^	30/50	30/50	32/48
Age (years)^a^	54.1 (7.8)	54.3 (7.8)	58.3 (7.5)
Height (m)^b^	174.0 (10.2)	172.3 (8.6)	172.7 (10.2)
Weight (kg)^c^	78.3 (15.8)	78.0 (16.1)	79.7 (15.8)
Mean pain intensity (NRS)^a^	3.08 (1.89)	2.94 (1.51)	3.10 (1.57)
RMDQ (number of items)^a^^,^^d^	6.8 (4.2)	5.0 (3.9)	5.1 (3.2)
Acute use of analgesics (n)^a^	31	26	18
No regular exercise (n)^a^	12	13	7

WB-EMS, whole-body electromyostimulation; WBV, whole-body vibration; CT, conventional back-strengthening training.

a Assessed by baseline questionnaire.

b Measured *via* stadiometer.

c Measured *via* bio-impedance analysis.

d Roland-Morris Disability Questionnaire: measured functional limitations due to low back pain and consists of a 24-point scale.

Written informed consent was obtained from all participants after giving comprehensive study instructions. After checking for eligibility, 240 participants were assigned to either a WB-EMS group (*n* = 80), a WBV group (*n* = 80), or a CT group (*n* = 80). The assignment was randomized and stratified according to basal pain intensity. In order to minimize effects of unspecific training loads, all participants were asked to refrain from any changes of their habitual physical activity behavior and were instructed to maintain their normal dietary intake before and during the study.

### Sample Size Estimation, Randomization, and Blinding Procedures

The required sample size was calculated with *R* statistics (R Development Core Team Vienna, Austria) and the *R* package “knitr” (Xie, 2015). Sample size estimation is based on a meta-analysis of individual patient data (Kemmler et al., 2017) and on results of a current meta-analysis by Searle et al. (2015). Based on these studies, a standardized mean difference (SMD) of 0.5 was assumed for the primary outcome MPI. As the primary question leads to two statistical tests, a Bonferroni correction was used accordingly with *α* = 0.05/2. Power was set at 0.8. On the basis of these assumptions, 78 subjects were needed for each group.

In three consecutive rounds per multicenter location from April 2017 to October 2019, a total number of 240 participants were randomly assigned into three groups in a balanced order (1:1:1) by drawing lots. The lots were placed in opaque plastic housings and stratified according to the basal numeric pain rating scale (NRS; 1–3, 4–6, and 7–10). The randomization was stopped after all three groups were sufficiently and equally filled in both multicenter locations, according to the initial sample size estimation. Neither participants nor researchers were able to know the allocation beforehand. After each balanced group allocation, the participants were informed about further study processes.

Due to organizational reasons, only research assistants and outcome assessors (blinded to group allocation) were blinded.

### Outcome Measures and Testing Procedures

The primary outcome measures were the changes in average low back pain intensity from baseline to 12-week follow-up, measured with a 4-week pain diary *via* daily self-report on a numeric pain rating scale (NRS 0–10; 0 = no pain, 10 = worst possible pain) at the same time of each day. The baseline pain intensity was recorded in the 4 weeks before the training intervention and the 12-week follow-up pain was recorded in the last 4 weeks of the training intervention.

Secondary outcomes were (a) changes in maximum isometric trunk extension and (b) changes in maximum isometric trunk flexion from baseline to 12-week follow-up, measured with an isometric strength testing machine (Back-Check® 607, Dr.Wolff, Arnsberg, Germany). Each participant performed three highly standardized isometric test attempts for trunk extension and flexion. The mean value out of the three tests was subsequently used for further analysis.

In both locations (Cologne and Erlangen), the same procedure, test equipment, and questionnaires were used. In each of the three consecutive rounds per location, the same researcher performed the testing.

### Intervention

Although some study results show that, especially for WBV training, individualized and personalized training programs should be used (Di Giminiani et al., 2015), this study chose general and recommended training programs that are also used by commercial and non-commercial providers. Thus, the study was able to maintain its claim that a real-world scenario with training programs actually used in practice was examined.

#### WB-EMS-Training and Conventional Back-Strengthening-Training

The intervention program of WB-EMS-Training and CT is described in detail elsewhere (Weissenfels et al., 2019). Briefly, participants of the WB-EMS group performed a total of 12 training sessions (TS), once a week over the 12-week intervention period. The total duration of a training session was 20 min, with a habituation phase in the first 4 weeks (12–20 min/TS). Each session contains of six trunk specific exercises with three sets and six repetitions. Stimulation parameters of WB-EMS were as following: bipolar, 85 Hz, 350 μs, 6 s stimulation, and 4 s rest. Surface electrodes (miha bodytec, Augsburg, Germany) were applied to the leg, arm, and trunk muscles. The EMS intensity was subjectively adjusted *via* the BORG CR10 (Borg, 1998) scale. Participants were supervised and instructed to train at a rate of perceived exertion between “strong” 5 and “very strong” 7.

Participants of the CT group performed a total of 12 training sessions once a week. The duration of a training session was 45 min: 15 min of aerobic warm up exercises and 30 min circuit training. Each circuit training consisted of 10 static or dynamic exercises for back strength/core stability. The exercises were performed twice, successively in a circuit training structure with 50 s of exercise followed by 25 s of rest. Participants were supervised and instructed to train at a rate of perceived exertion between “strong” 5 and “very strong” 7.

#### WBV-Training

Participants of the WBV group performed a total of 24 training sessions, twice a week over the 12-week intervention period. The total duration of a training session was 15 min. Each session contains of five exercises [(1) dynamic cable squats, (2) squats with arm extension, (3) calf raises, (4) static squats with arm movement, and (5) static cable squats with calf raises] with two sets and 5–8 repetitions. One minute of exercise was intermitted by 30 s of active rest. Exercises were performed with shoes in a standing position on a side-alternating vibration platform (Wellengang, Mühlacker, Germany). The frequency of the vibration ranged from 5 to 10 Hz and varied between the five exercises (Exercise 1: 5→6 Hz; Exercise 2: 7→8 Hz; Exercise 3: 10 Hz; Exercise 4: 8→10 Hz; Exercise 5: 8 Hz). The feet were placed shoulder-width at equal distance from the center of the platform corresponding to a peak-to-peak displacement of a maximum of 9 mm. Peak acceleration ranged from 0.45 g (5 Hz) to 1.81 g (10 Hz) as a function of frequency. Sixty seconds of oscillation were followed by 30 s of rest. During the active rests, the following exercises were performed: relaxed standing, hip swing, and hanging in the cable pull. Participants were instructed to train at a rate of perceived exertion between “strong” 5 and “very strong” 7.

### Statistical Analysis

Data were given as means or mean changes with SD. Statistical analyses were performed with the *R* statistics software package (R Development Core Team Vienna, Austria) in combination with a multiple imputation by Amelia II. The entire data set was multiply imputed using a 100 times imputation procedure. Normal distribution was graphically conducted *via* visual inspection of residuals for both primary and secondary endpoints (qq-plot). For the primary and secondary endpoint changes from baseline to follow-up, an intention-to-treat (ITT) analysis was used (packages “mice” and “miceadds”). A Welch 1-Way ANOVA was computed for significance testing (Allison, 2009). In case of significant results, pairwise Welch *t*-tests with adjusted values of *p* were conducted (Holm, 1979). The level of significance was set at *p* < 0.05.

Standardized mean differences (SMD) were calculated for the changes from baseline to follow-up. The magnitude of SMD was classified according to the following scale: 0–0.19 = negligible effect; 0.20–0.49 = small effect; 0.50–0.79 = moderate effect; and ≥0.80 = large effect (Cohen, 1988).

## Results

Twenty-seven participants had to terminate study participation due to individual reasons (e.g., diseases, injuries, and time; see Figure 1).

The attendance rate was high for all groups (WB-EMS: 92.0 ± 7.2%; WBV: 91.0 ± 7.0%; CT: 88.0 ± 8.0%).

### Mean Back Pain Intensity

**Figure 2:**
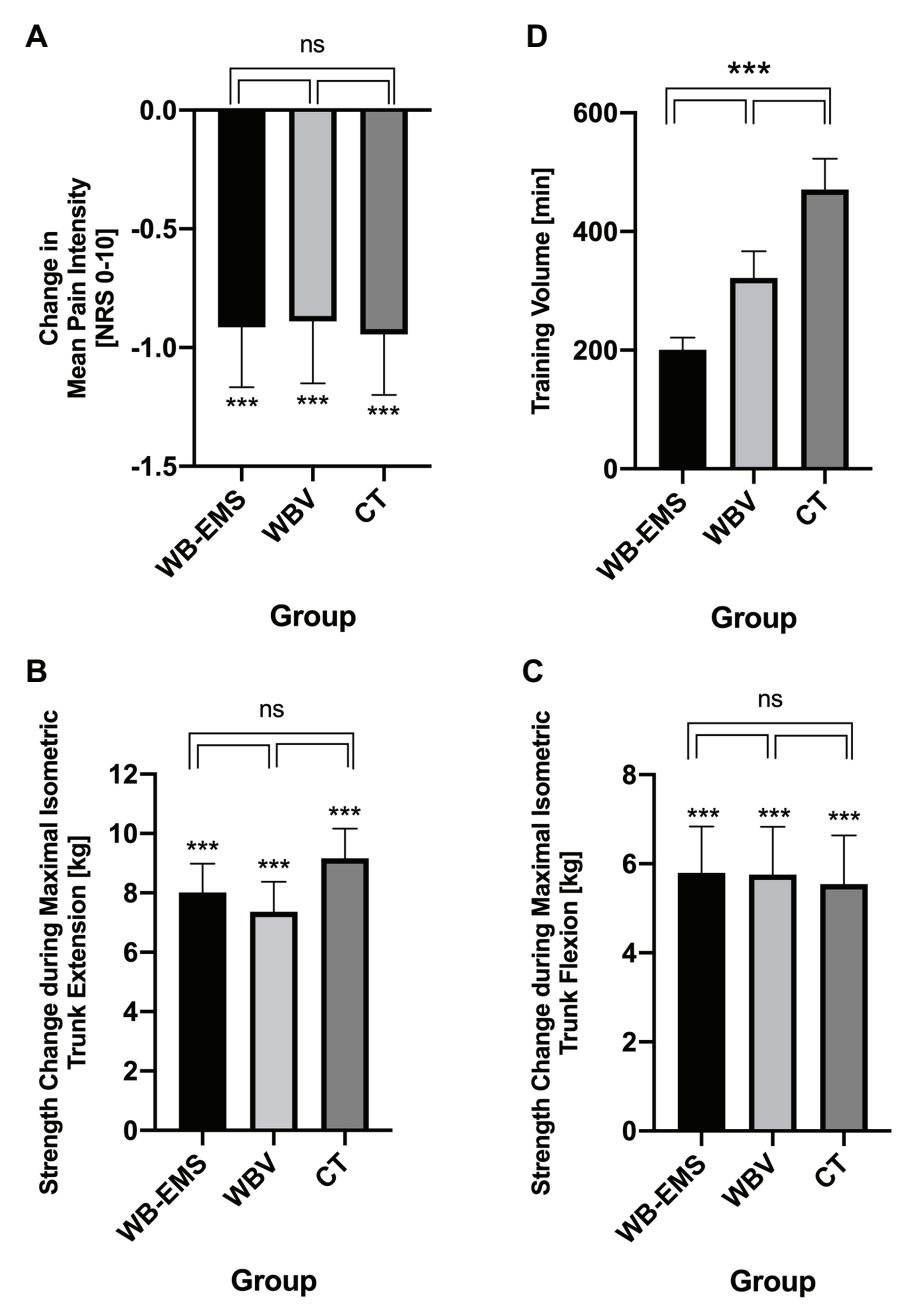
Mean changes in pain intensity **(A)**, maximal isometric trunk extension **(B)** and trunk flexion **(C)**, and training volume **(D)** of the three intervention groups. Values are presented as means ± SD. ***p < 0.001; ns, not significant.

Baseline values of the primary study endpoint mean pain intensity were as follows: WB-EMS 3.08 ± 1.89 NRS, WBV 2.94 ± 1.51 NRS, and CT 3.10 ± 1.57 NRS. There was a significant decline in all groups [WB-EMS: 29.7 ± 39.1% (SMD 0.50) vs. WBV: 30.3 ± 39.3% (SMD 0.57) vs. CT: 30.5 ± 39.6% (SMD 0.59); *p* < 0.001]. However, no significant intergroup effect could be observed (*p* = 0.934; *η*_p_^2^ = 0.002; Figure 2A).

Based on similar baseline values, 16% of participants in the WBV and WB-EMS group and 10% in the CT group reported no improvement or even worsening of MPI. Two participants (1 WBV and 1 WB-EMS) specified a relevant worsening (>2 NRS points) of MPI. Slight to moderate improvements (1–2 NRS points) in MPI were reported by 78% of the participants in the CT group, 70% in the WBV, and 68% in the WB-EMS intervention group. More prominent reductions (>2 NRS points) in MPI were recorded in 12% of participants in the CT group, 14% in the WBV group, and 16% in the WB-EMS group. Three participants, each in the WBV and WB-EMS groups, reported MPI reductions of more than 3 NRS points.

### Trunk Strength

Baseline values of maximum isometric trunk extension were as follows: WB-EMS 47.0 ± 18.6 kg, WBV 45.5 ± 17.3 kg, and CT 42.5 ± 16.5 kg. A significant increase in trunk extension strength was observed in all groups (extension: WB-EMS: 17.1 ± 25.5% vs. WBV: 16.2 ± 23.6% vs. CT: 21.6 ± 27.5%; *p* < 0.001). Baseline values of maximum isometric trunk flexion were as follows: WB-EMS 43.7 ± 18.2 kg, WBV 41.5 ± 17.7 kg, and CT 40.0 ± 19.8 kg. A significant increase in trunk flexion strength was also observed in all groups (flexion: WB-EMS: 13.3 ± 25.6% vs. WBV: 13.9 ± 24.0% vs. CT: 13.9 ± 25.4%; *p* < 0.001). However, no significant interaction effects could be observed for trunk extension (*p* = 0.475; *η*_p_^2^ = 0.009) and trunk flexion (*p* = 0.970; *η*_p_^2^ = 0.001; Figures 2B,C).

### Training Volume

Due to the different training protocols, the total training time differed significantly between the three intervention groups (WB-EMS: 200.9 ± 20.4 min vs. WBV: 321.5 ± 45.0 min vs. CT: 470.6 ± 52.3 min; Figure 2D).

### Confounders

During the intervention period, the number of participants with an acute intake of analgesics changed. In the WB-EMS group, it decreased from 31 to 11, in the WBV group from 26 to 18 and in the CT group from 18 to 11. Although additional treatments were prohibited during the intervention, 26 participants (WB-EMS: 9; WBV: 9; and CT: 8) started one (e.g., physiotherapy, massage, and acupuncture). In contrast, 11 participants stopped a previously started treatment (WB-EMS: 2; WBV: 4; and CT: 5).

## Discussion

This study investigated the effects of three different 12-week lasting training interventions (WB-EMS vs. WBV vs. CT) on mean changes of back pain intensity and selected strength parameters of the trunk muscles in patients with CNLBP. To the best of our knowledge, and in addition to the study of Weissenfels et al. (2019), this is the first randomized controlled intervention study that compares the alternative training technologies WB-EMS and WBV with a conventional back-strengthening exercise program.

The main findings of this study indicate that (a) WB-EMS and WBV reduce pain intensity and increase trunk strength and (b) both training technologies provide comparable training adaptations than CT, but are significantly more time-efficient. Of note, with about 30% reductions on the NRS 0–10 scale in all the groups, changes in chronic pain intensity can be considered as clinically important (Farrar et al., 2001).

With regard to our primary endpoint “back pain intensity,” a recent meta-analysis from Searle et al. (2015) shows positive results in favor of conventional exercise. With significant small to moderate effects, subgroup analysis showed that the most effective treatments in this meta-analysis were interventions with strength/resistance exercises (SMD = 0.50) and coordination/stabilization exercises (SMD = 0.47). With moderate effects in all three training groups of this study, the reductions of low back pain are very similar. It should be noted that in terms of volume and frequency, the mean of the included studies of the meta-analysis and the present study are also very similar. Strength/resistance studies with high effects on pain reduction, for example, by Steele et al. (2013; SMD=1.69) or Kell et al. (Kell and Asmundson, 2009; SMD=2.14) differ especially in the training intensity with additional loads up to 70–80% of the One-Repetition-Maximum. Such high loads were not used in the present study, which is why these results are difficult to compare.

The systematic review by Wang et al. (2020) revealed that there are few trials of good quality (PEDro scale 6–8) that assessed the efficacy of WBV on CNLBP (del Pozo-Cruz et al., 2011; Kaeding et al., 2017; Wang et al., 2019). All interventions lasted for 12 weeks with 2–3 training sessions a week. The results of the study by del Pozo-Cruz et al. (2011; −24.1% in VAS) and Wang et al. (2019; −34.6% in VAS) show that the pain reductions are also very similar than those of the WBV group in this study that are exactly in between (−30.3% in NRS). The differently chosen stimulation patterns in these studies (frequency: 10–30 Hz, amplitude 0–6 mm, and duration of sets 1–5), therefore, do not seem to significantly influence the results on pain reduction as much as might be assumed.

In the field of WB-EMS, there are only one meta-analysis of individual patient data (Kemmler et al., 2017) and one controlled non-randomized clinical intervention study (Konrad et al., 2020) focusing on low back pain. The included studies of the meta-analysis show a wide range of training duration from 14 weeks to 12 months with different training volume and frequency. With a mean pain intensity decrease of 16.9% on a 7-level scale (Kemmler et al., 2017), the changes are slightly lower than in the current study. The differences of age (72.0 ± 7.1), training frequency (1.5 sessions/week) and the unspecific assessment tools for back pain in the meta-analysis might explain the differences between the higher results of this study. The results of the clinical intervention study confirm our findings. A WB-EMS training program, conducted over 24 weeks with a comparable training volume (1x/week, 20 min) showed significant and clinically important pain reductions by 2 points on the NRS 0–10 scale (Konrad et al., 2020). The slightly greater pain reductions can be explained by the longer training period (12 vs. 24 weeks) and the higher basal pain values.

With regard to trunk strength serving as secondary endpoint, there are scarce intervention studies on the alternative training technologies verifying this endpoint in CNLBP patients. Only one WB-EMS study including elderly women (75 ± 4 years) with sarcopenia investigating maximal isometric trunk extension (Kemmler et al., 2014). Despite a longer intervention phase (12 months) and a higher training frequency (1.5 sessions/week), the results (+10.1 ± 12.7%) were lower than in the present study. However, the participants of that study were much older and suffered from diseases that might confound the effects. Generally, when considering the significant increases in trunk strength, it should be noted that the exercises in WB-EMS and WBV were not specifically designed to improve strength.

Time restrictions are one of the main reasons for CNLBP patients not to train (Korsch et al., 2016). In terms of time expenditure, the alternative training methods, especially WB-EMS, show a significant lower training volume (training frequency × training duration) with nearly the same results in pain reduction and in strength improvements. Thus, the participants of the WB-EMS group needed only 43% of the adjusted total training time of the CT group and 62% of the WBV group.

Regarding future research, some limitations and weaknesses of the present study need to be addressed. (1) The subjects of the present study showed a relatively low to moderate mean pain intensity at baseline. As a result, the potential for change may be considerably less than with high basal pain intensities. It is also unclear whether the present results can be generalized to patients with high levels of low back pain. (2) Although the change in covariates influencing the primary study endpoint was consistently queried, especially the acute intake of analgesics and the inclusion of additional treatments, and the change in pain medication showed no differences between the groups, it is possible that this factor could slightly influence our results. (3) The assumption of non-specific back pain was merely made on the basis of questionnaires and self-reporting. No detailed written clinical diagnoses by clinicians were employed to verify the self-reports. Therefore, this information could be notably biased by a combination of several factors. (4) Although our own data showed that the measurements of the secondary study outcome trunk strength were highly reliable, it needs to be mentioned that adequate validity studies are lacking. This point holds particularly true for our testing procedure of maximal isometric trunk extension and flexion testing in CNLBP patients. Despite a high level of test standardization, testing in the standing position must be considered as suboptimal as it allows the M.gluteii, the leg extensors and small abdominal muscles to contribute to fore generation. (5) In terms of training frequency, duration, and the general setting, the training conditions of the three interventions differed significantly and were further not individualized to the study participants. These differences are closely oriented to the training practice of commercial and non-commercial providers. However, slight variations of these training protocols could lead to different effects on study outcomes such as mean pain intensity. Especially in the areas of WB-EMS and WBV, there is a lack of comparative studies for the development of optimized training protocols for a large number of health-relevant outcomes. (6) The focus of this intervention study was to assess behavioral/phenomenal outcomes. However, the underlying mechanisms of pain reduction of the different training methods remain unclear. Thus, further studies should examine the neural/physiological/structural modulations that occur as a result of training, especially WB-EMS and WBV, in terms of pain reduction in CNLBP patients.

## Conclusion

All three training programs significantly reduce MPI of patients with CNLBP. The alternative training technologies WB-EMS and WBV are comparably effective than the conventional back-specific training program in order to reduce low back pain. However, the reductions in pain and the increase in isometric trunk strength could be achieved at notably different training volumes.

Depending on individual factors such as time availability and personal preferences, patients can choose between different training programs for pain treatment. WB-EMS and WBV offer effective and time-efficient alternatives for CNLBP patients to reduce substantial disease burden on a personal, community, and financial level.

## Data Availability Statement

The raw data supporting the conclusions of this article will be made available by the authors, without undue reservation.

## Ethics Statement

The studies involving human participants were reviewed and approved by Ethical Committee of the Friedrich-Alexander University Erlangen-Nürnberg (application no. 224_15b). The patients/participants provided their written informed consent to participate in this study.

## Author Contributions

FM carried out the study in the multicenter location Cologne, participated in study design and interpretation of data, and drafted the manuscript. AW carried out the study in the multicenter location Erlangen, participated in study design and interpretation of data, and reviewed the manuscript. NW and UD participated in study design, accompanied study conduction in Cologne, and reviewed the manuscript. SS participated in study design, accompanied study conduction in Erlangen, and reviewed the manuscript. MK performed the statistical analysis. LD supported study conduction in Cologne and helped to write the manuscript. WK and HK conceived the study, participated in the design, coordination, and interpretation of data, acquired the research funds, and reviewed the manuscript. All authors have read and approved the final version of the manuscript.

### Conflict of Interest

The authors declare that the research was conducted in the absence of any commercial or financial relationships that could be construed as a potential conflict of interest.

## References

[ref1] Abraham I. Killackey-Jones B. (2002). Lack of evidence-based research for idiopathic low back pain: the importance of a specific diagnosis. Arch. Intern. Med. 162, 1442–1444. 10.1001/archinte.162.13.1442, PMID: 12090876

[ref2] Allison P. D. (2009). Missing data [Nachdr.]. Thousand Oaks, Calif: Sage Publ.

[ref3] Ardigò L. P. Iacono A. D. Zagatto A. M. Bragazzi N. L. Kuvacic G. Bellafiore M.. (2018). Vibration effect on ball score test in international vs. national level table tennis. Biol. Sport 35, 329–334. 10.5114/biolsport.2018.78051, PMID: 30765917 PMC6358531

[ref4] Bo Andersen L. Wedderkopp N. Leboeuf-Yde C. (2006). Association between back pain and physical fitness in adolescents. Spine 31, 1740–1744. 10.1097/01.brs.0000224186.68017.e0, PMID: 16816772

[ref5] Borg G. (1998). Borg’s perceived exertion and pain scales. Champaign, IL: Human Kinetics.

[ref6] Citko A. Górski S. Marcinowicz L. Górska A. (2018). Sedentary lifestyle and nonspecific low back pain in medical personnel in North-East Poland. Biomed. Res. Int. 2018:1965807. 10.1155/2018/1965807, PMID: 30271778 PMC6151221

[ref7] Cohen J. (1988). Statistical power analysis for the behavioral sciences. Hillsdale, New Jersey: Lawrence Erlbaum Associates.

[ref8] Costa L. O. P. Maher C. G. Latimer J. Hodges P. W. Herbert R. D. Refshauge K. M.. (2009). Motor control exercise for chronic low back pain: a randomized placebo-controlled trial. Phys. Ther. 89, 1275–1286. 10.2522/ptj.20090218, PMID: 19892856

[ref9] Critchley D. J. Ratcliffe J. Noonan S. Jones R. H. Hurley M. V. (2007). Effectiveness and cost-effectiveness of three types of physiotherapy used to reduce chronic low back pain disability: a pragmatic randomized trial with economic evaluation. Spine 32, 1474–1481. 10.1097/BRS.0b013e318067dc26, PMID: 17572614

[ref10] Cuesta-Vargas A. I. García-Romero J. C. Arroyo-Morales M. Diego-Acosta A. M. Daly D. J. (2011). Exercise, manual therapy, and education with or without high-intensity deep-water running for nonspecific chronic low back pain: a pragmatic randomized controlled trial. Am. J. Phys. Med. Rehabil. 90, 526–534. 10.1097/PHM.0b013e31821a71d0, PMID: 21765272

[ref11] del Pozo-Cruz B. Hernández Mocholí M. A. Adsuar J. C. Parraca J. A. Muro I. Gusi N. (2011). Effects of whole body vibration therapy on main outcome measures for chronic non-specific low back pain: a single-blind randomized controlled trial. J. Rehabil. Med. 43, 689–694. 10.2340/16501977-0830, PMID: 21687923

[ref12] Di Giminiani R. Masedu F. Padulo J. Tihanyi J. Valenti M. (2015). The EMG activity-acceleration relationship to quantify the optimal vibration load when applying synchronous whole-body vibration. J. Electromyogr. Kinesiol. 25, 853–859. 10.1016/j.jelekin.2015.09.004, PMID: 26443890

[ref13] Farrar J. T. Young J. P. LaMoreaux L. Werth J. L. Poole R. M. (2001). Clinical importance of changes in chronic pain intensity measured on an 11-point numerical pain rating scale. Pain 94, 149–158. 10.1016/S0304-3959(01)00349-9, PMID: 11690728

[ref14] Filipovic A. Grau M. Kleinöder H. Zimmer P. Hollmann W. Bloch W. (2016). Effects of a whole-body electrostimulation program on strength, sprinting, jumping, and kicking capacity in elite soccer players. J. Sports Sci. Med. 15, 639–648., PMID: 27928210 PMC5131218

[ref15] Filipovic A. Kleinöder H. Dörmann U. Mester J. (2011). Electromyostimulation—a systematic review of the influence of training regimens and stimulation parameters on effectiveness in electromyostimulation training of selected strength parameters. J. Strength Cond. Res. 25, 3218–3238. 10.1519/JSC.0b013e318212e3ce, PMID: 21993042

[ref16] Fischer M. Vialleron T. Laffaye G. Fourcade P. Hussein T. Chèze L.. (2019). Long-term effects of whole-body vibration on human gait: a systematic review and meta-analysis. Front. Neurol. 10:627. 10.3389/fneur.2019.00627, PMID: 31316447 PMC6611385

[ref17] Hanel J. Owen P. J. Held S. Tagliaferri S. D. Miller C. T. Donath L.. (2020). Effects of exercise training on fear-avoidance in pain and pain-free populations: systematic review and meta-analysis. Sports Med. 50, 2193–2207. 10.1007/s40279-020-01345-1, PMID: 32946074

[ref18] Holm S. (1979). A silmple sequentially rejective multiple test procedure. Scand. J. Stat. 6, 65–70. 10.2307/4615733

[ref19] Hoy D. Bain C. Williams G. March L. Brooks P. Blyth F.. (2012). A systematic review of the global prevalence of low back pain. Arthritis Rheum. 64, 2028–2037. 10.1002/art.34347, PMID: 22231424

[ref20] Ishak N. A. Zahari Z. Justine M. (2017). Kinesiophobia, pain, muscle functions, and functional performances among older persons with low back pain. Pain Res. Treat. 2017:3489617. 10.1155/2017/3489617, PMID: 28634547 PMC5467352

[ref21] Jackson J. K. Shepherd T. R. Kell R. T. (2011). The influence of periodized resistance training on recreationally active males with chronic nonspecific low back pain. J. Strength Cond. Res. 25, 242–251. 10.1519/JSC.0b013e3181b2c83d, PMID: 20093971

[ref22] Kaeding T. S. Karch A. Schwarz R. Flor T. Wittke T.-C. Kück M.. (2017). Whole-body vibration training as a workplace-based sports activity for employees with chronic low-back pain. Scand. J. Med. Sci. Sports 27, 2027–2039. 10.1111/sms.12852, PMID: 28185300

[ref23] Kell R. T. Asmundson G. J. G. (2009). A comparison of two forms of periodized exercise rehabilitation programs in the management of chronic nonspecific low-back pain. J. Strength Cond. Res. 23, 513–523. 10.1519/JSC.0b013e3181918a6e, PMID: 19209082

[ref24] Kemmler W. Bebenek M. Engelke K. von Stengel S. (2014). Impact of whole-body electromyostimulation on body composition in elderly women at risk for sarcopenia: the Training and ElectroStimulation Trial (TEST-III). Age 36, 395–406. 10.1007/s11357-013-9575-2, PMID: 23949160 PMC3889893

[ref25] Kemmler W. Shojaa M. Steele J. Berger J. Fröhlich M. Schoene D.. (2021). Efficacy of whole-body electromyostimulation (WB-EMS) on body composition and muscle strength in non-athletic adults. A systematic review and meta-analysis. Front. Physiol. 12:640657. 10.3389/fphys.2021.640657, PMID: 33716787 PMC7952886

[ref26] Kemmler W. Weissenfels A. Bebenek M. Fröhlich M. Kleinöder H. Kohl M.. (2017). Effects of whole-body electromyostimulation on low back pain in people with chronic unspecific dorsal pain: a meta-analysis of individual patient data from randomized controlled WB-EMS trials. Evid. Based Complement. Alternat. Med. 2017:8480429. 10.1155/2017/8480429, PMID: 29234437 PMC5664316

[ref27] Kemmler W. Weissenfels A. Willert S. Shojaa M. von Stengel S. Filipovic A.. (2018). Efficacy and safety of low frequency whole-body electromyostimulation (WB-EMS) to improve health-related outcomes in non-athletic adults. A systematic review. Front. Physiol. 9:573. 10.3389/fphys.2018.00573, PMID: 29875684 PMC5974506

[ref28] Konrad K. L. Baeyens J.-P. Birkenmaier C. Ranker A. H. Widmann J. Leukert J.. (2020). The effects of whole-body electromyostimulation (WB-EMS) in comparison to a multimodal treatment concept in patients with non-specific chronic back pain-A prospective clinical intervention study. PLoS One 15:e0236780. 10.1371/journal.pone.0236780, PMID: 32822361 PMC7446884

[ref29] Korsch S. Herbold D. Wiezoreck M. Geigner B. Beddies A. Worringen U.. (2016). Förderfaktoren, barrieren und barrierenmanagement zur umsetzung gesundheitsförderlicher verhaltensweisen von rehabilitanden mit chronischem rückenschmerz—eine qualitative analyse [Promoting factors, barriers and barrier management to the implementation of health-promoting behavior among rehabilitative patients with chronic low back pain—a qualitative analysis]. Rehabilitation 55, 210–216. 10.1055/s-0042-106844 27529297

[ref30] Lai C.-C. Tu Y.-K. Wang T.-G. Huang Y.-T. Chien K.-L. (2018). Effects of resistance training, endurance training and whole-body vibration on lean body mass, muscle strength and physical performance in older people: a systematic review and network meta-analysis. Age Ageing 47, 367–373. 10.1093/ageing/afy009, PMID: 29471456

[ref31] Lüning Bergsten C. Lundberg M. Lindberg P. Elfving B. (2012). Change in kinesiophobia and its relation to activity limitation after multidisciplinary rehabilitation in patients with chronic back pain. Disabil. Rehabil. 34, 852–858. 10.3109/09638288.2011.624247, PMID: 22214399

[ref32] Manchikanti L. Singh V. Falco F. J. E. Benyamin R. M. Hirsch J. A. (2014). Epidemiology of low back pain in adults. Neuromodulation 17(Suppl. 2), 3–10. 10.1111/ner.12018, PMID: 25395111

[ref33] Mannion A. F. Müntener M. Taimela S. Dvorak J. (2001). Comparison of three active therapies for chronic low back pain: results of a randomized clinical trial with one-year follow-up. Rheumatology 40, 772–778. 10.1093/rheumatology/40.7.772, PMID: 11477282

[ref34] Nassif H. Brosset N. Guillaume M. Delore-Milles E. Tafflet M. Buchholz F.. (2011). Evaluation of a randomized controlled trial in the management of chronic lower back pain in a French automotive industry: an observational study. Arch. Phys. Med. Rehabil. 92, 1927.e4–1936.e4. 10.1016/j.apmr.2011.06.029, PMID: 22133239

[ref35] Padulo J. Di Giminiani R. Dello Iacono A. Zagatto A. M. Migliaccio G. M. Grgantov Z.. (2016). Lower arm muscle activation during indirect-localized vibration: the influence of skill levels when applying different acceleration loads. Front. Physiol. 7:242. 10.3389/fphys.2016.00242, PMID: 27378948 PMC4909772

[ref36] Padulo J. Di Giminiani R. Ibba G. Zarrouk N. Moalla W. Attene G.. (2014a). The acute effect of whole body vibration on repeated shuttle-running in young soccer players. Int. J. Sports Med. 35, 49–54. 10.1055/s-0033-1345171, PMID: 23780902

[ref37] Padulo J. Filingeri D. Chamari K. Migliaccio G. M. Calcagno G. Bosco G.. (2014b). Acute effects of whole-body vibration on running gait in marathon runners. J. Sports Sci. 32, 1120–1126. 10.1080/02640414.2014.889840, PMID: 24576194

[ref38] Rapoport J. Jacobs P. Bell N. R. Klarenbach S. (2004). Refining the measurement of the economic burden of chronic diseases in Canada. Chronic Dis. Can. 25, 13–21., PMID: 15298484

[ref39] Searle A. Spink M. Ho A. Chuter V. (2015). Exercise interventions for the treatment of chronic low back pain: a systematic review and meta-analysis of randomised controlled trials. Clin. Rehabil. 29, 1155–1167. 10.1177/0269215515570379, PMID: 25681408

[ref40] Shaughnessy M. Caulfield B. (2004). A pilot study to investigate the effect of lumbar stabilisation exercise training on functional ability and quality of life in patients with chronic low back pain. Int. J. Rehabil. Res. 27, 297–301. 10.1097/00004356-200412000-00007, PMID: 15572993

[ref41] Steele J. Bruce-Low S. Smith D. Jessop D. Osborne N. (2013). A randomized controlled trial of limited range of motion lumbar extension exercise in chronic low back pain. Spine 38, 1245–1252. 10.1097/BRS.0b013e318291b526, PMID: 23514876

[ref42] Vincent H. K. Vincent K. R. Seay A. N. Conrad B. P. Hurley R. W. George S. Z. (2014). Back strength predicts walking improvement in obese, older adults with chronic low back pain. PM R 6, 418–426. 10.1016/j.pmrj.2013.11.002, PMID: 24211698 PMC4013252

[ref43] Wang X.-Q. Gu W. Chen B.-L. Wang X. Hu H.-Y. Zheng Y.-L.. (2019). Effects of whole-body vibration exercise for non-specific chronic low back pain: an assessor-blind, randomized controlled trial. Clin. Rehabil. 33, 1445–1457. 10.1177/0269215519848076, PMID: 31099264

[ref44] Wang W. Wang S. Lin W. Li X. Andersen L. L. Wang Y. (2020). Efficacy of whole body vibration therapy on pain and functional ability in people with non-specific low back pain: a systematic review. BMC Complement. Med. Ther. 20:158. 10.1186/s12906-020-02948-x, PMID: 32460819 PMC7251707

[ref45] Weissenfels A. Teschler M. Willert S. Hettchen M. Fröhlich M. Kleinöder H.. (2018). Effects of whole-body electromyostimulation on chronic nonspecific low back pain in adults: a randomized controlled study. J. Pain Res. 11, 1949–1957. 10.2147/JPR.S164904, PMID: 30288089 PMC6160275

[ref46] Weissenfels A. Wirtz N. Dörmann U. Kleinöder H. Donath L. Kohl M.. (2019). Comparison of whole-body electromyostimulation versus recognized back-strengthening exercise training on chronic nonspecific low back pain: a randomized controlled study. Biomed. Res. Int. 2019:5745409. 10.1155/2019/5745409, PMID: 31687394 PMC6794965

[ref47] Xie Y. (2015). Dynamic documents with R and knitr. 2nd Edn. Boca Raton, FL: CRC Press.

